# Biomarkers of Gemtuzumab Ozogamicin Response for Acute Myeloid Leukemia Treatment

**DOI:** 10.3390/ijms21165626

**Published:** 2020-08-06

**Authors:** Laurène Fenwarth, Elise Fournier, Meyling Cheok, Thomas Boyer, Fanny Gonzales, Sylvie Castaigne, Nicolas Boissel, Juliette Lambert, Hervé Dombret, Claude Preudhomme, Nicolas Duployez

**Affiliations:** 1 UMR 9020–UMR-S 1277–Canther–Cancer Heterogeneity, Plasticity and Resistance to Therapies, Institut de Recherche contre le Cancer de Lille, University Lille, CNRS, Inserm, CHU Lille, F-59000 Lille, France; elise.fournier@chru-lille.fr (E.F.); meyling.cheok@inserm.fr (M.C.); fanny.gonzales@chru-lille.fr (F.G.); claude.preudhomme@chru-lille.fr (C.P.); nicolas.duployez@chru-lille.fr (N.D.); 2 Laboratory of Hematology, CHU Amiens, F-80054 Amiens, France; boyer.thomas@chu-amiens.fr; 3 Department of Hematology, CH Versailles, F-78157 Le Chesnay, France; scastaigne@ch-versailles.fr (S.C.); jlambert@ch-versailles.fr (J.L.); 4 Adolescent and Young Adult Hematology Unit, Hôpital Saint-Louis, AP-HP, Université de Paris, F-75010 Paris, France; nicolas.boissel@aphp.fr; 5 Department of Hematology, Hôpital Saint-Louis, AP-HP, Université de Paris, F-75010 Paris, France; herve.dombret@aphp.fr

**Keywords:** acute myeloid leukemia, gemtuzumab ozogamicin, biomarkers, CD33, FLT3, therapy

## Abstract

Gemtuzumab ozogamicin (GO, Mylotarg^®^) consists of a humanized CD33-targeted antibody-drug conjugated to a calicheamicin derivative. Growing evidence of GO efficacy in acute myeloid leukemia (AML), demonstrated by improved outcomes in CD33-positive AML patients across phase I to III clinical trials, led to the Food and Drug Administration (FDA) approval on 1 September 2017 in CD33-positive AML patients aged 2 years and older. Discrepancies in GO recipients outcome have raised significant efforts to characterize biomarkers predictive of GO response and have refined the subset of patients that may strongly benefit from GO. Among them, CD33 expression levels, favorable cytogenetics (t(8;21), inv(16)/t(16;16), t(15;17)) and molecular alterations, such as *NPM1*, *FLT3*-internal tandem duplications and other signaling mutations, represent well-known candidates. Additionally, in depth analyses including minimal residual disease monitoring, stemness expression (LSC17 score), mutations or single nucleotide polymorphisms in GO pathway genes (*CD33*, *ABCB1*) and molecular-derived scores, such as the recently set up CD33_PGx6_Score, represent promising markers to enhance GO response prediction and improve patient management.

## 1. Introduction

Standard of care for acute myeloid leukemia (AML) has long been based on chemotherapy combinations with or without hematopoietic stem cell transplantation (HSCT). Despite efforts in supportive care improvement, 5-year overall survival (OS) of adult patients with AML remains at 30–40% [1]. Over the past years, significant advances have been made in understanding the AML mutational landscape, identifying leukemic cells and characterizing their intrinsic properties leading to the development of new drugs, among which eight have been approved by the Food and Drug Administration (FDA) for the treatment of AML between 2017 and 2019. Notably, gemtuzumab ozogamicin (GO, CMA-676, Mylotarg^®^) is a humanized cluster of differentiation 33 (CD33)-targeted antibody-drug conjugated to a calicheamicin derivative, a natural antitumor antibiotic. CD33 antigen represents a hallmark of myeloid leukemic blasts, widely expressed in AML patients. Several clinical studies have highlighted the clinical benefit of GO on patient outcome. GO stands for the first antibody drug conjugate approved by the FDA. Enhanced knowledge about the GO metabolic pathway at both cellular and molecular levels has raised and improved understanding on GO response biomarkers.

**Figure 1:**
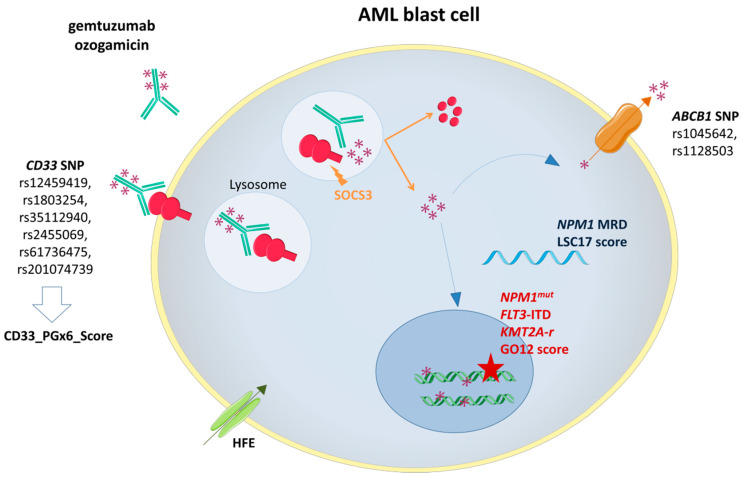
Gemtuzumab ozogamicin (GO) mechanism of action and biomarkers of response. SOCS3: Suppressor Of Cytokine Signaling 3; *ABCB1*: ATP-binding cassette subfamily B member 1 gene; *NPM1*^mut^: Nucleophosmin 1 gene mutation; *FLT3*-ITD: FMS-Like Tyrosine Kinase 3 Internal Tandem Duplication; *KMT2A*-r: Lysine Methyltransferase 2A rearrangement.

After a brief review about the mechanism of action of GO and its efficacy across successive clinical trials, this review will discuss the biomarkers predicting GO response (Figure 1).

## 2. Gemtuzumab Ozogamicin

### 2.1. CD33: The Target Antigen

The CD33 antigen is a 67 kD single chain transmembrane glycoprotein that belongs to the sialic-acid-binding immunoglobulin-like lectins family (Siglecs) [2]. The *CD33* gene, located on chromosome 19q13.4, is composed of eight exons. Exons 1 and 2 encode for the amino-terminal V-set signal peptide, an immunoglobulin-like domain mediating the sialic-acid binding, exons 3 and 4 encode the C2-set domain, and exon 5 encodes the transmembrane domain. The intracytoplasmic domain, encoded by exons 6, 7a and 7b, comprises two tyrosine-based inhibitory signaling motifs (Y340 and Y358) which, upon phosphorylation, provide docking sites for the Src homology-2 domain-containing tyrosine phosphatases (SHP) and the suppressor of cytokine signaling 3 (SOCS3) [3,4,5]. In turn, SHP-1 and SHP-2 dephosphorylate CD33 and negatively regulate other surrounding receptors [3]. SOCS3 competes with SHP-1/2 for CD33 binding and recruits the Elongin B/C-Cul2/Cul5-SOCS-box protein E3 ubiquitin ligase leading to the proteasomal degradation of CD33 and SOCS3 [6].

CD33 is a differentiation antigen especially expressed among myeloid progenitors, while it is not expressed by normal hematopoietic stem cells [7]. AML originates from clonal evolution of driver and cooperative genetic alterations in multipotent CD34^+^/CD33^−^ stem cells and/or in committed CD34^+^/CD33^+^ myeloid progenitors [8,9,10]. Previous studies have shown that CD33 was expressed on leukemic blasts in 85% to 90% of AML patients [11,12]. Collectively, these data raised a huge interest to consider CD33 as potent and selective therapeutic target in AML.

### 2.2. Mechanism of Action

GO consists of a recombinant humanized immunoglobulin G4 kappa CD33-targeted antibody (hP67.6) covalently linked to the semi-synthetic antitumor antibiotic of the enediyne family, the N-acetyl gamma calicheamicin, via the acid-labile hybrid 4-(4′-acetylphenoxy)butanoic acid linker [13]. After binding to the CD33 antigen, the complex GO-CD33 is rapidly internalized [14]. In the cytoplasm, this complex is routed in the lysosome. Under the acidic environment of the lysosome, the butanoic acid linker is hydrolyzed, releasing the toxic moiety of the GO. The calicheamicin derivative is reduced by the glutathione into a highly reactive species which induces simple- and double-stranded DNA breaks, leading to DNA-damage [15,16,17]. Downstream, the DNA repair pathway is activated through the ataxia-telangiectasia mutated (ATM)/ataxia-telangiectasia and Rad3-related (ATR) and the DNA-dependent protein kinase pathways [18,19]. In turn, ATM and ATR proteins phosphorylate Chk1 and Chk2 proteins, which eventually results in G2/M cell cycle arrest. The DNA-dependent pathway activation mediates DNA repair through H2AX phosphorylation. Hence, cells defective in ATM, DNA-dependent protein kinase or genes coding for the non-homologous end joining repair are hypersensitive to calicheamicin [16,20]. However, the predominant downstream pathway following the ATM/ATR activation is the mitochondrial apoptotic pathway mediated by the B-cell lymphoma 2 (Bcl-2) family proteins Bax and Bak which releases the cytochrome-c and eventually activates caspases 9 and 3. This pro-apoptotic pathway acts independently of the tumor protein 53 (TP53) and Fas-Associated protein with Death Domain (FADD)-signaling pathways [21,22]. Data from a phase II trial suggest that Bcl-2 antisense (Oblimersen sodium) may enhance the pro-apoptotic pathway in patients treated concomitantly with GO [23].

### 2.3. Clinical Data

Successive clinical trials have demonstrated the anti-leukemic activity of GO and its clinical benefit on patient outcome (Table 1).

#### 2.3.1. GO Administered as Monotherapy

In a phase I dose escalation trial, 40 adult patients with relapsed/refractory CD33-positive AML received GO until a maximum dose of 9 mg/m^2^ saturating almost all AML CD33-binding sites (92.2%) with an acceptable safety profile [24]. Similarly, a phase I trial undertaken in 29 children with relapsed/refractory AML, showed the tolerability and the efficacy of GO (overall response rate [ORR], 28%) [37].

Three phase II trials assessed the safety and the efficacy of GO as a single agent given at 9 mg/m^2^ on day 1 and day 14 in adult AML patients experiencing first relapse. A total of 30% of the patient population achieved complete remission (CR)/complete remission without platelet recovery (CRp) [25]. Based on these results, GO was granted FDA accelerated approval on 17 May 2000 as a monotherapy for CD33-positive AML patients older than 60 years of age, experiencing first relapse and unfit for intensive treatment [41]. Of note, final analysis revealed that this GO schedule was associated with frequent grade 3 and 4 hematological toxicities (profound neutropenia and thrombocytopenia) and liver toxicities (veno-occlusive disease) [42].

The European Organization for Research and Treatment of Cancer-Gruppo Italiano Malattie Ematologiche Maligne dell’Adulto (EORTC-GIMEMA) AML-19 trial is a sequential phase II/III trial that first determined the best GO induction regimen (GO administered as monotherapy, 6 mg/m^2^ on day 1 plus 3 mg/m^2^ on day 8 versus [vs.] GO 3 mg/m^2^ on days 1, 3 and 5) before undertaking the phase III trial comparing GO to best supportive care in previously untreated AML patients older than 61 years unfit for intensive chemotherapy. The GO schedule 6 mg/m^2^ on day 1 plus 3 mg/m^2^ on day 8 led to a higher rate of disease non-progression, defined as the rate of patients in CR/CRp or in stable disease at the end of induction course (63% vs. 38%), and was retained for the phase III [43]. In the subsequent phase III, first-line GO monotherapy significantly improved OS compared to best supportive care (1-year OS: 24.3% vs. 9.7%, hazard ratio [HR]: 0.69, 95% CI: 0.53–0.90, *p* = 0.005) [36].

#### 2.3.2. GO Administered in Combination with Intensive Chemotherapy

A phase III randomized trial was undertaken by the Southwest Oncology Group (SWOG, S0106 trial) to further confirm the clinical benefit of the addition of GO (6 mg/m^2^ on day 4) to the standard ‘3 + 7’ induction regimen, associating daunorubicin and cytarabine. Patients allocated to the GO arm received lower dose of daunorubicin (45 mg/m^2^ vs. 60 mg/m^2^ in the control arm) in order to balance toxicities [29]. However, the interim analysis revealed higher rate of fatal induction toxicities in the GO arm (5.5% vs. 1.4%, *p* = 0.0062) leading to the premature end of the study and withdrawal from the market on 21 June 2010. Final data analysis of the S0106 trial failed to demonstrate any clinical benefit of the addition of GO neither on relapse-free survival (RFS) nor on OS (GO arm vs. non GO arm: 5-year RFS: 43% vs. 42%, *p* = 0.40; 5-year OS: 46% vs. 50%, *p* = 0.85).

In an attempt to reduce GO toxicities, a dose-finding trial assessed the addition of GO 3 mg/m^2^ vs. 6 mg/m^2^ to intensive chemotherapy regimens. The addition of GO 6 mg/m^2^ was not feasible due to hepatotoxicity and delayed hematopoietic recovery. However, 3 mg/m^2^ GO appeared effective and safe [44]. This study led to two randomized phase III trials, addressing the clinical benefit of adding GO 3 mg/m^2^ to the induction regimen in younger adults (Medical Research Council (MRC) AML15 trial) and in older patients (National Cancer Research Institute (NCRI) AML16 trial) [28,30]. In these two trials, the addition of GO showed an improved OS in older patients, and in younger adults with favorable-risk AML. Given the rapid re-expression on the CD33 antigen after GO exposure [14,17], a phase II trial addressed the efficacy of fractionated GO (3 mg/m^2^ on days 1, 4 and 7) in adults with relapsed AML [26]. Fractionated GO administered as monotherapy led to a CR/CRp rate of 33% with a good safety profile, in particular, no grade 3 or 4 liver toxicity. When combining fractionated GO to chemotherapy, 65% to 75% of patients achieved CR/CRp [27,45]. Based on these encouraging results of lowering and fractionating doses of GO, the randomized phase III trial Acute Leukemia French Association (ALFA)-0701 compared the clinical benefit of low fractionated doses of GO in addition to the standard intensive chemotherapy regimen in newly diagnosed CD33-positive AML patients, aged 50 to 70 years old [32]. In the experimental arm, patients were administered fractionated doses of GO 3 mg/m^2^, during induction (on days 1, 4 and 7) and consolidation courses (on day 1 of each course). Event-free survival (EFS) was remarkably improved in the GO arm (median EFS, assessed by the blinded independent review: 13.6 vs. 8.5 months, HR: 0.66, 95% CI: 0.49–0.89, *p* = 0.006) whilst median OS did not significantly differ between the two arms (HR: 0.81, 95% CI: 0.60–1.09, *p* = 0.16) [33]. These results led the FDA to approve GO for newly diagnosed CD33-positive AML in adults and for relapsed or refractory CD33-positive AML in patients aged 2 years and older on 1 September 2017. GO received the European Medicines Agency’s (EMA) marketing authorization on 19 April 2018 for the treatment of de novo CD33-positive AML patients aged 15 years and above as frontline therapy in combination with daunorubicin and cytarabine.

Corroborating these results, a meta-analysis including 3325 patients from five open-label randomized phase III controlled trials (MRC AML15, NCRI AML16, SWOG S0106, GOELAMS-AML 2006 IR and ALFA-0701 [28,29,30,31,32]) highlighted the benefit of the addition of GO on the risk of relapse (RR) and on OS (RR: OR: 0.81, 95% CI: 0.73–0.90, *p* = 0.0001; 5-year OS: OR: 0.90, 95% CI: 0.82–0.98, *p* = 0.01) [46].

More recently, a phase III trial, the NCRI AML17 trial, evaluated the impact of GO dosing 3 vs. 6 mg/m^2^ combined with intensive chemotherapy in previously untreated AML patients. Among the 788 included patients, increased GO dosing (6 mg/m^2^) did not improve neither response rate nor patient outcome (OS: HR: 1.10, 95% CI: 0.90–1.34, *p* = 0.3; RFS: HR: 1.11, 95% CI: 0.91–1.35, *p* = 0.3) [34].

In the pediatric population, phase II studies from the Children’s Oncology Group (COG, AAML00P2 and AAML03P1) demonstrated the benefit of the addition of GO to chemotherapy [38,39]. In the subsequent phase III trial AAML0531, AML patients aged 0 to 29 years with newly diagnosed AML were randomly assigned to a five-course chemotherapy regimen alone or combined with two doses of GO 3 mg/m^2^ (on day 6 during induction course 1, and on day 7 during intensification course 2). Among the 1022 evaluable patients, GO recipients experienced better EFS (3-year EFS: 53.1% vs. 46.9%, HR: 0.83, 95% CI: 0.70–0.99, *p* = 0.04) but no improved OS (3-year OS: 69.4% vs. 65.4%, HR: 0.91, 95% CI: 0.74–1.13, *p* = 0.39) [40]. Based on these results, the FDA extended the indication of GO to newly diagnosed CD33-positive AML patients aged one month and older, on June 16, 2020.

## 3. Biomarkers

### 3.1. CD33 Expression on AML Cells

As previously described, CD33 is widely expressed in AML patients (>80%, [25]). However, the CD33 expression level on leukemic cells is heterogeneous, varying more than 2 log-fold among AML patients [47,48]. In the pediatric AAML03P1 cohort, high expression of CD33 is associated with poor outcome in multivariate analysis [47,49]. In vitro studies demonstrated that GO-induced cytotoxicity was highly dependent on cell surface expression of CD33 and higher CD33 expression levels correlated with an increase of GO binding to CD33 antigenic sites and thus enhanced clearance of AML blasts [14,50]. Remarkably, good responders among GO recipients express higher mean of CD33 expression levels inversely correlated with a low ATP-binding cassette subfamily B-member 1 (ABCB1) expression which mediates drug efflux [51]. Among the 825 patients from the pediatric AAML0531 trial, GO improved EFS in patients with high CD33 expression (quartiles (Q), Q2-Q4, GO vs. no-GO, 5-year EFS: 53% vs. 41%, *p* = 0.005), whereas patients with low CD33 expression (Q1) did not benefit from the addition of GO to the induction chemotherapy (GO vs. no-GO, 5-year EFS: 53% vs. 58%, *p* = 0.456) [48]. By contrast, in the adult population no interaction on survival was observed between CD33 expression quartiles and GO in non-core binding factor (CBF)-AML patients [52]. However, GO reduced the RR of adult patients with Q4-CD33 expression (HR: 0.63, 95% CI: 0.35–1.12). Higher CD33 expression have been observed in patients displaying mutations in Nucleophosmin 1 (*NPM1)* gene or FMS-like tyrosine kinase 3 internal tandem duplications (*FLT3*-ITD) and is further detailed in the corresponding section [47,48,52,53,54,55]. In a retrospective analysis performed on 200 adult patients from the ALFA-0701 trial, and considering CD33 expression as a binary variable defined by a 70% cutoff, GO improved EFS and RFS of patients with high CD33 expression even after adjustment for cytogenetics and *NPM1*/*FLT3*-ITD mutations [55]. Thus CD33 expression appears, as expected, as an important pre-treatment biomarker for GO response.

### 3.2. Prognostic Impact of the Cytogenetic Alterations on GO Efficacy

The benefit of GO has been widely observed in the non-adverse cytogenetic-risk groups [28,32,36,46,56]. In a meta-analysis, the addition of GO was strongly associated with a survival benefit in patients from good (acute promyelocytic leukemia [APL] excluded) and intermediate cytogenetic groups (absolute survival benefit at 6 years: + 20.7%; OR: 0.47, 95% CI: 0.31–0.73, *p* = 0.0006; + 5.7%; OR: 0.84, 95% CI: 0.75–0.95, *p* = 0.005, respectively) [46]. Conversely, in the adverse cytogenetic group, in which the CD33 expression level is usually lower, GO failed to improve OS (absolute survival benefit at 6 years: + 2.2%, OR: 0.99, 95% CI: 0.83–1.18, *p* = 0.9).

CBF-AMLs correlate with a low CD33 expression [52]. It is assumed that the first event, t(8;21)/inv(16)/t(16.16), initiates very early in preleukemic CD33^-^ cells [57]. By contrast, subsequent driver events, such as tyrosine kinase mutations leading to the proliferative AML phenotype occur when the cells express CD33 [58]. Hence, GO could eradicate the proliferative clone and spare CD33^-^ preleukemic cells. Additionally, GO efficacy in CBF-AML may be explained by a high sensitivity of CBF-AML blasts to calicheamicin [9,59]. A phase II trial assessed the efficacy of the FLAG induction regimen, including fludarabine, cytarabine and filgrastim, combined with GO 3 mg/m^2^, at induction day 1, and post-remission courses 1 and 2 day 1 (FLAG-GO) as frontline therapy in adult CBF-AML patients. Among the 45 enrolled patients, the FLAG-GO regimen was associated with a high remission rate (95%), and 3-year OS and RFS of 78% and 85% respectively [60]. A recent study assessed the benefit of the FLAG-GO regimen compared to the association FLAG and idarubicin regimen (FLAG-Ida). The FLAG-GO regimen was associated with a higher molecular response rate (76% vs. 42%, *p* = 0.002) and an improved 5-year RFS (87% vs. 68%, *p* = 0.02), but not 5-year OS (*p* = 0.7) compared to the FLAG-Ida regimen [61].

The standard of care for APL treatment relies on all-trans retinoic acid (ATRA) plus or minus arsenic trioxide (ATO)-based regimen [62,63]. However, APL blast cells express the CD33 antigen in nearly 100% of APL patients along with a low ABCB1 expression and offer the opportunity of a new treatment option for APL patients [64,65,66]. Administered as a single-agent or combined with ATRA, GO improved response rate of APL patients [67,68,69,70]. Interestingly, in vitro studies have reported the efficacy of GO in ATRA- or arsenic trioxide (ATO)-resistant APL cell lines which translated into remission achievement in clinical trials [65,71]. The combination of ATRA plus ATO with or without GO appeared safe and effective, with a CR rate of 90%, and 81% in high-risk patients [72]. These results were further confirmed in the phase III UK-NCRI AML17 trial [70]. In high-risk APL patients treated with ATRA, the addition of GO plus ATO was as effective as the adjunction of idarubicine (5-year OS: 84% vs. 100%, *p* = 0.453) [73]. Recently, the SWOG assessed the efficacy of ATRA plus ATO with GO combination in high-risk APL patients in a phase II trial (SWOG S0535). Among the 70 evaluable patients, 86% achieved CR and the 3-year OS and 3-year RFS were 86% and 91%, respectively [74]. Hence, this chemotherapy-free combination appears as a relevant option in high-risk APL patient management.

11q23/lysine methyltransferase 2A (*KMT2A)* rearrangements are recurrent cytogenetic alterations in AML, more commonly identified in children. These rearrangements correlate with elevated CD33 expression levels on leukemic cells [48,75]. Several reports have highlighted the anti-leukemic effect of GO in relapsed/refractory KMT2A-rearranged AML [76,77]. In the COG AAML0531 trial, 215 patients harbored a 11q23/*KMT2A* rearrangement. In this patient population, patients treated with GO plus chemotherapy experienced a significantly higher EFS than those treated with chemotherapy alone (5-year EFS: 48% vs. 28%, *p* = 0.002), although 5-year OS did not reach significant difference across treatment arms (5-year OS: 64% vs. 53%, *p* = 0.053) [78].

### 3.3. Prognostic Impact of the Molecular Profile on GO Efficacy

Aside from favorable cytogenetic alterations, GO seems to benefit patients with *NPM1* or *FLT3* mutations.

*NPM1* mutations are identified in 25% to 35% of AML patients and more frequently in cytogenetically normal AML (45%–60%) [79]. As previously mentioned, patients with *NPM1* mutations display higher CD33 expression levels [53,54,55]. In the ALFA-0701 trial, the subset analyses pointed out the benefit of the addition of GO on 2-year EFS, RFS and OS in *NPM1*-mutated patients [32]. Recently, the prospective, randomized phase III trial Acute Myeloid Leukemia Study Group (AMLSG) 09-09 addressed the clinical benefit of the addition of GO to induction (3 mg/m^2^ on day 1) and consolidation chemotherapy (3 mg/m^2^ on day 1 of the first consolidation cycle) in adult patients with *NPM1*-mutated AML eligible to receive intensive chemotherapy [80]. Among the 588 included patients, 292 were assigned to the GO arm and 296 to the standard arm. GO did not improve 2-year EFS in this trial (HR: 0.83, 95% CI: 0.65–1.04, *p* = 0.10). In patients achieving CR/CR with incomplete hematologic recovery, GO significantly decreased the cumulative incidence of relapse (HR: 0.66, 95% CI: 0.49–0.88, *p* = 0.005). Interestingly, GO improved 2-year EFS of *FLT3* wild-type, but not *FLT3*-ITD mutated patients (HR: 0.72, 95% CI: 0.56–0.95 vs. HR: 1.53, 95% CI: 0.95–2.48, respectively; interaction test, *p* = 0.002).

*FLT3*-ITD mutations are found in approximately 20% of AML patients, and are associated with high expression of CD33 and impaired outcome [79]. The addition of GO has demonstrated improved OS, EFS and RFS in adult AML patients with *FLT3*-ITD mutations [32,56]. In a retrospective analysis of *FLT3*-ITD mutated patients from the COG AAML03P1 and AAML0531 trials, the addition of GO was associated with a decreased RR (37% vs. 59%, *p* = 0.02) [81]. Among the subset of patients who underwent hematopoietic stem cell transplantation (HSCT) in first CR, this effect was even stronger, prior exposure to GO was associated with a reduced cumulative incidence of relapse (22% vs. 56%, *p* = 0.003). Patients displaying a high allelic ratio (> 0.4) experienced a lower RR of relapse when GO was administered prior to HSCT (15% vs. 53%, *p* = 0.007). By contrast, in the adult cohorts from the MRC AML15 and NCRI AML16 trials, GO did not improve clinical outcome of *FLT3*-ITD mutated AML patients. However, in these trials GO was administered as a single dose while fractionated doses of GO were administered in the ALFA-0701 and the COG AAML03P1 and AAML0531 trials.

Mutational profile of AML has been widely deciphered by high-throughput sequencing technologies over the past decade [82]. Mutations have become strong prognostic factors and have been integrated in the latest European LeukemiaNet (ELN) 2017 risk stratification [83]. A retrospective analysis from the ALFA-0701 showed a benefit of the addition of GO on EFS in patients from the ELN favorable-risk (HR: 0.54, 95% CI: 0.30–0.98, *p* = 0.04) and intermediate-risk groups (HR: 0.57, 95% CI: 0.33–1.00, *p* = 0.05), but not in patients from the ELN adverse-risk group (HR: 0.93, 95% CI: 0.61–1.43, *p* = 0.74) [84]. In particular, considering mutations by functional group as previously described [82], GO predominantly improved EFS of patients harboring signaling mutations, (HR: 0.43, 95% CI: 0.28–0.65) [84]. These mutations were associated with higher CD33 expression levels.

### 3.4. Prognostic Impact of Minimal Residual Disease (MRD)

AML prognosis highly depends on pretreatment markers such as cytogenetic and molecular alterations. These prognostic factors have been integrated in the latest ELN 2017 risk classification and guide HSCT decision [83]. Growing evidence has suggested the prognostic impact of persisting leukemic cells assessed by the minimal disease monitoring after induction even in patients achieving morphological CR [85,86,87,88,89,90,91]. Different cytometric or molecular markers have been evaluated to monitor MRD [92]. In the pediatric AML02 trial, patients were allocated to either chemotherapy alone, chemotherapy plus GO, or GO alone depending on MRD levels, assessed by flow cytometry [93]. Among patients with positive MRD, 13 out of 17 reached MRD negativity after GO administration alone, and 13 out of 29 had negative MRD after GO plus chemotherapy [94]. In the NCRI-AML16 trial, MRD measured by flow cytometry accounted for an independent prognostic factor for patient outcome. However, this trial failed to demonstrate a significantly higher proportion of MRD negativity measured by flow cytometry among patients receiving GO compared to the control arm (57% vs. 48%, *p* = 0.18) [85].

*NPM1* mutation and Wilms’ tumor 1 *(WT1)* gene overexpression represent valuable prognostic molecular markers to assess MRD by real-time quantitative polymerase chain reaction (RQ-PCR) [89,95,96,97,98,99,100,101]. In the ALFA-0701 study, the addition of GO to standard induction regimen increased *NPM1* MRD negativity proportion both at the end of induction and at the end of treatment (39% vs. 7%, *p* = 0.006; and 91% vs. 61% *p* = 0.028, respectively) [97]. By contrast, no impact on *WT1* MRD has been observed when adding GO to standard induction regimen neither after induction nor after end of treatment (MRD negativity: 75% vs. 65%, *p* = 0.29; 82% vs. 80%, *p* = 1, respectively). The lower sensitivity of *WT1* MRD compared to *NPM1* MRD may have accounted for this discrepancy [97].

These encouraging results of MRD monitoring in patients treated with GO gave rise to consider MRD as a surrogate endpoint for patient outcome. To date, several MRD-directed trials are currently investigating GO benefit [102].

### 3.5. GO and Stemness Signature

Different studies support the critical role of leukemic stem cells (LSC) in AML maintenance. LSC are characterized by intrinsic properties of cell cycle quiescence, self-renewal and increased drug efflux which confer chemotherapy resistance [8,103,104]. A recent study based on five independent AML cohorts (n = 908 patients) set up the LSC17 score, derived from a 17-gene expression signature for LSC [105]. This score stands for a strong prognostic factor in AML. Interestingly, in the ALFA-0701 trial, the addition of GO correlated with improved outcome in patients with low but not high LSC17 score (EFS: HR: 0.42, *p* = 0.001; RFS: HR: 0.53, *p* = 0.03). Hence, the LSC17 score appears as relevant biomarker to predict GO benefit in AML patients [105].

Patients harboring normal karyotype and *NPM1* mutations without *FLT3*-ITD are assimilated to a low molecular risk (LMR). Interestingly, the GO12 score, derived from 12 GO pathway genes accurately identified LMR/LMR-like patients that may benefit from GO across 5 independent AML cohorts (n = 1188 patients; area under the curve: 80.8%) [106].

#### 3.5.1. *CD33* Single Nucleotide Polymorphisms

Recent studies have addressed the relationship between *CD33* genotype and GO efficacy. A pivotal retrospective study from the St Jude (AML02 trial) found out a single nucleotide polymorphism (SNP) in the splice enhancer region of the *CD33* gene exon 2, rs12459419 (C > T; Ala14Val) that affected response to GO, as measured by flow cytometric MRD [107]. This SNP resulted in *CD33* exon 2 skipping, leading to a shorter *CD33* isoform lacking the immunoglobulin-like V-set domain which is the epitope for GO and for the P67.6-CD33 antibody, used for CD33-expression determination by immunophenotyping [108,109]. In patients displaying TT genotype, median CD33 expression was significantly lower than those with CT or CC genotype (TT vs. CT vs. CC: 44.8% vs. 97.4% vs. 152.2%, *p* < 0.001) [110]. These first results were further confirmed in AML patients aged 0 to 29 years from the AAML0531 trial [111]. Among the 816 patients genotyped for the SNP rs12459419, 51%, 39% and 10% of the patients had CC, CT, TT genotype, respectively. A benefit of the addition of GO was demonstrated on both RR and RFS only in patient with CC genotype. A recent similar study undertaken in younger adults with AML (13–69 years) from the randomized MRC AML15 and NCRI AML17 trials showed a similar distribution of CC, CT and TT genotypes (47%, 44%, 9%, respectively). However, this study failed to demonstrate any benefit of GO on OS and on RFS in the different genotype subgroups [112]. Likewise, the prognostic value of the *CD33* splice site genotype was evaluated in patients receiving an alternative CD33-targeting antibody-drug conjugate, the vadastuximab talirine (SGN-CD33A) administered as monotherapy or in combination with hypomethylating agents, in a cohort of 20 adult patients (mean age: 69.8 years, range: 27.5–82.6 years) with AML [113]. Genotyping analysis of *CD33* SNP rs12459419 revealed the following distribution of the CC/CT/TT genotypes: 50%/40%/10%. Similarly to the previous study led in adult patients, *CD33* splice site genotype did not impact patient outcome neither in OS (*p* = 0.923) nor in EFS (*p* = 0.683). Differences in trial designs including age range of inclusion and GO dosing may explain these discrepancies. The ABCB1-mediated drug efflux, which is higher in older patients, may have encountered for such differences between pediatric and adult populations [51,113,114,115].

Genotyping studies of *CD33* SNPs have identified five other SNP such as rs1803254(G > C; 3′UTR), rs35112940(G > A; Arg304Gly), rs2455069(A > G; Arg69Gly), rs61736475(Ser305Pro) and rs201074739 (CCGG deletion) which may modulate GO anti-leukemic effect [116]. A reduced RR in GO recipients was observed in patients displaying the following genotypes: rs1803254 GG (*p* = 0.009), rs35112940 GG (*p* < 0.001), rs2455069 GG (*p* = 0.005), rs61736475 TT (*p* = 0.002), and rs201074739 CCGG/CCGG (*p* = 0.002).

Interestingly, the CD33_PGx6_Score—a composite score derived from six *CD33* SNP of prognostic significance (rs12459419, rs2455069, rs201074739, rs35112940, rs61736475 and rs1803254)—has been recently set up to assess the impact of *CD33* genotype on CD33 expression and GO response among 938 patients with de novo AML, aged 0–29 years [116]. CD33_PGx6_Score of 0 or higher was associated with higher CD33 expression levels and improved RFS and reduced RR in patients treated in GO arm (GO vs. no-GO arm, 5-year RFS: 62.5% vs. 46.8%, *p* = 0.008; 5-year RR: 28.3% vs. 49.9%, *p* < 0.001). By contrast, the addition of GO showed no improvement on patient outcome when the score was less than 0.

#### 3.5.2. Prognostic Impact of ABCB1

Despite its pro-apoptotic effects, free calicheamicin may also be a substrate of the ABCB1 transporter (also known as permeability glycoprotein (Pgp) and multi-drug resistance protein (MDR1)) and to a lesser degree, the multidrug resistance-associated protein 1 (MRP1 or ABCC1), but not the breast cancer resistance protein (BCRP) [117,118,119]. Hence, ABCB1 and MRP1 may pump calicheamicin out of the cell before exerting its cytotoxic activity and ultimately compromise GO efficacy.

ABCB1 is expressed in 58% of AML patients and its expression on blasts cells varies from 19% to 75% [118,120]. ABCB1 expression strongly correlates with response to GO, and higher expression level of ABCB1 stands for an independent poor prognostic factor in OS and EFS [51,117,120,121]. Importantly, in a retrospective study from three phase II trials [25,42,117], the expression of CD33 was inversely correlated with the ABCB1 drug efflux activity. However, after adjusting for CD33 expression, ABCB1 was still associated with outcome (*p* < 0.001) [51].

Interestingly, a comprehensive analysis of ABCB1 demonstrated that ABCB1 expression was shown to correlate with low white blood cell count and high expression of the following genes: *CD34*, *BAALC*, *CD7* and *CD200* [120]. Additionally, ABCB1 activity seems to be linked to the absence of *FLT3*-ITD and *NPM1* mutations.

A recent study has assessed the clinical impact of *ABCB1* genotype among 942 patients from the COG-AAML0531 trial [122]. GO recipients displaying CT or TT genotype for the SNP rs1045642 (C > T; Ile1145Ile) had improved outcomes compared to those with CC genotype (CT or TT vs. CC, 5-year EFS: *p* = 0.022; 5-year RR: *p* = 0.007) as a result of an increased accumulation of calicheamicin.

#### 3.5.3. SOCS3 Methylation

By binding to the CD33, SOCS3 induces the proteasomal degradation of the CD33-SOCS3 complex [6]. SOCS3 expression was suggested to modulate anti-CD33 antibodies response [123].

Analysis of the methylation status of the *SOCS3* CpG islands was associated with a trend of improved response rate and OS in patients with *SOCS3* hypermethylation (ORR: 86% vs. 56%, *p* = 0.17; OS: 25.1 months vs. 10.3 months; HR: 0.29%, 95% CI: 0.06–1.32, *p* = 0.09) [124].

#### 3.5.4. HFE Mutations

*HFE* mutations are associated with higher risk of cancer. Interestingly, GO improved patient outcome among heterozygote *HFE* mutated patients compared to wild type patients, probably related to an impaired CD33 internalization [125].

## 4. Conclusions

Given its high expression on AML blasts, CD33 antigen represents an attractive target in AML. Different clinical trials have confirmed the anti-leukemic activity of GO in CD33-positive AML cells and have shown improved outcome in AML patients. Over the past years, flow cytometry, cytogenetics, and molecular approaches, including sequencing technologies, MRD monitoring, and genotyping studies of *CD33* and *ABCB1* SNPs have offered a comprehensive analysis of promising biomarkers for GO response. Collectively, these improvements have helped to refine the subset of patients that may benefit from GO and improve patient management. Increasing knowledge of the molecular alterations in AML paves the way to new combinatory regimens that may enhance GO efficacy. Hence, ongoing trials are evaluating the feasibility and the efficacy of combining GO to FLT3-ITD inhibitors (NCT03900949, NCT04385290, NCT04293562) and Bcl-2 inhibitors (NCT04070768, NCT04070768).

## Figures and Tables

**Table 1:** Overview of the main clinical trials evaluating GO efficacy

Trial Acronym	Dates of Recruitement	Phase	Patient Population	Median Age of Patients in Years (Range)	Evaluable Patients	GO Dosing	Treatment Plan	Outcomes	Ref.
**Adult Trials**									
	NA	I	Relapsed/refractory AML patients	54 (24–73)	40	Escalating doses, 0.25 to 9 mg/m^2^	Single arm trial, GO administered as single agent	ORR: 8/40 patients (20%)	Sievers 1999 [24]
	1997–1999	II	AML patients in first relapse	61 (22–84)	142	9 mg/m^2^, 2 doses recommended (max. 3 doses), with at least 14 days between 2 doses	Single arm trial, GO administered as single agent	ORR: 42/142 patients (30%), CR rate: 16%, CRp rate: 13%	Sievers 2001 [25]
Mylofrance-1	2005	II	De novo AML in first relapse	64 (22–80)	57	Fractionated doses: 3 mg/m^2^ on days 1, 4 and 7 of the first course	Single arm trial, GO administered as single agent in induction, followed by cytarabine-based consolidation	ORR: 19/57 (33%), CR rate: 15/57 (26%), CRp: 4/57 (7%)	Taksin 2007 [26]
Mylofrance-2	2006–2007	I/II	De novo AML in first relapse	60 (40–70)	20	Fractionated doses: 3 mg/m^2^ on days 1, 4 and 7 of the first course	Single arm trial, GO combined with DA (DA dosing finding)	ORR: 13/20 patients (65%), CR rate: 11/20 patients (55%), CRp rate: 2/20 patients (10%)	Farhat 2012 [27]
MRC AML15	2002–2006	III	De novo/secondary AML	50 (15–71)	1113	3 mg/m^2^ on day 1 of course 1 +/− on day 1 of the course 3	Randomization at induction and at consolidation. Induction regimen (DA or ADE or FLAG-Ida) +/− GO. Consolidation regimen (MACE or MidAC or high-dose cytarabine) +/− GO	GO- vs. no GO-arm: CR, 82% vs. 83%, OR: 1.04, 95% CI: 0.76–1.42, *p* = 0.8; 5-year OS, 43% vs. 41%, HR: 0.92, 95% CI: 0.79–1.08, *p* = 0.3; 5-year RFS: 39% vs. 35%, HR: 0.87, 95% CI: 0.73–1.02, *p* = 0.09	Burnett 2011 [28]
SWOG S0106	2004–2009	III	De novo AML	47 (18–60)	595	6 mg/m^2^ on day 4; additional 3 doses of GO, 5 mg/m^2^ for patients in CR after consolidation	Randomized trial, GO plus modified DA (daunorubicin, 45 mg/m^2^/d, day 1 to day 3; cytarabine, 100 mg/m^2^/d, day 1 to day 7) vs. standard DA (daunorubicin, 60 mg/m^2^/d, day 1 to day 3; cytarabine, 100 mg/m^2^/d, day 1 to day 7)	DA + GO vs. DA alone: ORR: 76% vs. 74%, *p* = 0.36; CR rate: 69% vs. 70%, *p* = 0.59; 5-year RFS: 43% vs. 42%, *p* = 0.40; 5-year OS: 46% vs. 50%, *p* = 0.85	Petersdorf 2013 [29]
NCRI AML16	2006–2010	III	De novo/secondary AML and high-risk MDS	67 (51–84)	1115	3 mg/m^2^ on day 1 of the first course	Randomized trial: DA or daunorubicin/clofarabine +/− GO	GO- vs. no GO-arm: ORR: 70% vs. 68%, OR: 0.88, 95% CI: 0.68–1.13, *p* = 0.3; 3-year OS: 25% vs. 20%; HR: 0.87, 95% CI: 0.76–1.00, *p* = 0.05; 3-year RFS: 21% vs. 16%, HR: 0.84, 95%CI: 0.71–0.99, *p* = 0.04	Burnett 2012 [30]
GOELAMS-AML 2006 IR	2007–2010	III	De novo AML patients with intermediate cytogenetic risk	50 (18–60)	238	6 mg/m^2^ on day 4 of the induction and on day 4 of the first consolidation course	Randomized trial: intensive induction regimen (DA) +/− GO, consolidation (MidAC) +/− GO, +/− HSCT	GO- vs. no-GO-arm: CR rate: 91.6% vs. 86.5%, *p* = NS; 3-year OS: 53% vs. 46%, *p* = NS; 3-year EFS: 51% vs. 33%, *p* = NS. In non HSCT recipients, GO vs. no GO-arm: 3-year EFS: 53.7% vs. 27%, *p* = 0.0308	Delaunay 2011 [31]
ALFA-0701	2008–2010	III	De novo AML	62 (50–70)	271	3 mg/m^2^ on days 1, 4, and 7 of induction and on day 1 of each of the subsequent two consolidation courses	Randomized trial: DA +/− GO	GO- vs. no-GO-arm: ORR: 81.5% vs. 73.5% (*p* = 0.15) (CR: 70.4% vs. 69.9%; CRp:11.1% vs. 3.7%); median EFS: 13.6 months vs. 8.5 months, HR: 0.66, 95% CI: 0.49–0.89, *p* = 0.006; median OS: 27.5 months vs. 21.8 months, HR: 0.81, 95% CI: 0.60–1.09, *p* = 0.16	Castaigne 2012, Lambert 2019 [32,33]
UK NCRI AML17	2009–2011	III	De novo or secondary AML and high-risk MDS	50 (0–81)	788	3 mg/m^2^ vs. 6 mg/m^2^ on day 1 of induction	Randomized trial: GO 3 vs. 6 mg/m^2^ + combined with ADE vs. DA	GO 3 mg/m^2^ vs. 6 mg/m^2^: ORR: 89% vs. 86%, HR: 1.34, 95%CI:0.88–2.04, *p* = 0.17; (CR rate 82% vs. 76%, OR: 1.46, 95%CI: 1.04–2.06, *p* = 0.03); 4-year OS: 50% vs. 47%, HR: 1.10, 95% CI: 0.90–1.34, *p* = 0.3; 4-year RFS: 44% vs. 38%, HR: 1.11, 95% CI: 0.91–1.35, *p* = 0.3	Burnett 2016 [34]
EORTC-GIMEMA AML-17	2002–2007	III	De novo/secondary AML	67 (60–75)	472	3 mg/m^2^ for 2 doses, on days 1 and 15 of induction, 3 mg/m^2^ on the first day of consolidation	Randomized trial: intensive chemotherapy (MICE induction) +/− GO	GO vs. no-GO-arm: ORR: 45% vs. 49%; OR: 0.86, 95% CI, 0.6–1.23, *p* = 0.46; OS: HR: 1.20, 95% CI: 0.99–1.45, *p* = 0.07; RFS: HR: 1.08, 95% CI: 0.81–1.44, *p* = 0.61	Amadori 2013 [35]
EORTC-GIMEMA AML-19	2004–2013	III	De novo/secondary AML unfit for intensive chemotherapy	77 (62–88)	237	6 mg/m^2^ on day 1 and 3 mg/m^2^ on day 8, +/−2 mg/m^2^/month for up to 8 doses	Randomized trial: GO alone vs. BSC	GO- vs. BSC-arm: median OS: 4.9 months vs. 3.6 months, HR: 0.69, 95% CI: 0.53–0.90, *p* = 0.005	Amadori 2016 [36]
**Pediatric trials**									
	1999–2002	I	Relapsed/refractory AML patients	12 (1–16)	29	Escalating doses, 6 to 9 mg/m^2^	Single arm trial, GO administered as single agent	ORR: 8/29 patients (28%); CR rate: 14%; CRp rate: 14%)	Arceci et al. 2005 [37]
COG-AAML00P2	2002–2006	II	Refractory de novo AML or newly diagnosed secondary AML	11.5 (0.8–19.8)	45	2 to 3 mg/m^2^	Non randomized multi-arm trial, GO + cytarabine + mitoxantrone (arm A) vs. GO+ cytarabine+ L-asparaginase (arm B)	Arm A vs. arm B: ORR: 55% vs. 40%, *p* = NS; 1-year EFS: 55% vs. 21.8%, *p* = NS; 1-year OS: 64.6% vs. 45.0% *p* = NS	Aplenc 2008 [38]
COG-AAML03P1	2003–2005	II	Newly diagnosed de novo AML	9.5 (0.07–21.6)	340	3 mg/m^2^ on day 6 of course 1 and day 7 of course 4	Single arm trial, GO combined with intensive chemotherapy	CR rate: 83.1%; 3-year OS: 66%; 3-year EFS: 53%	Cooper 2012 [39]
COG-AAML0531	2006–2010	III	Newly diagnosed de novo AML	9.7 (0–29)	1022	3 mg/m^2^ on day 6 of induction course 1, and on day 7 of intensification course 2	Randomized trial, GO +/− standard chemotherapy	GO- vs. no-GO arm: CR rate: 88.3% vs. 85.1, *p* = 0.15; 3-year EFS: 53.1% vs. 46.9%, HR: 0.83, 95% CI: 0.70–0.99, *p* = 0.04; 3-year OS: 69.4% vs. 65.4%; HR: 0.91, 95% CI: 0.74–1.13, *p* = 0.39	Gamis 2014 [40]

COG: Children’s Oncology Group; MDS: myelodysplastic syndrome; CR: Complete Remission; CRp: all criteria for CR without the full recovery of platelets count; ORR: overall response rate (CR+CRp); DA: daunorubicin plus cytarabine; DAE: cytarabine, daunorubicin, and etoposide; FLAG-Ida: fludarabine, cytarabine, granulocyte colony-stimulating factor, and idarubicin; MACE: amsacrine, cytarabine and etoposide; MidAC: mitoxantrone and cytarabine; MICE: mitoxantrone, cytarabine, and etoposide; BSC: best supportive care, HSCT: hematopoietic stem cell transplantation; NA: Not available; NS: not significant.

## Abbreviations

ABC	ATP-Binding Cassette
ABCB1	ATP-Binding Cassette Subfamily B Member 1
ALFA	Acute Leukemia French Association
AML	Acute Myeloid Leukemia
AMLSG	Acute Myeloid Leukemia Study Group
APL	Acute Promyelocytic Leukemia
ATM	Ataxia-Telangiectasia Mutated
ATR	Ataxia-Telangiectasia and Rad3-Related
ATRA	All-Trans Retinoic Acid
BCRP	Breast Cancer Resistance Protein
Bcl-2	B-Cell Lymphoma 2
BSC	Best Supportive Care
CBF	Core-Binding Factor
CD	Cluster Of Differentiation
COG	Children’s Oncology Group
CR	Complete Remission
CRp	Complete Remission Without Platelet Recovery
DA	Daunorubicin Plus Cytarabine
DAE	Cytarabine, Daunorubicin, And Etoposide
ECS	Elongin B/C-Cul2/Cul5-Socs-Box Protein
EFS	Event-Free Survival
ELN	European LeukemiaNet
EMA	European Medicines Agency
EORTC	European Organization For Research And Treatment Of Cancer
FADD	Fas-Associated Protein With Death Domain
FDA	Food And Drug Administration
FLAG-Ida	Fludarabine, Cytarabine, Granulocyte Colony-Stimulating Factor, and Idarubicin
*FLT3*-ITD	FMS-Like Tyrosine Kinase 3 Internal Tandem Duplication
GIMEMA	Gruppo Italiano Malattie Ematologiche Maligne Dell’adulto
GO	Gemtuzumab Ozogamicin
HR	Hazard Ratio
HSCT	Hematopoietic Stem Cell Transplantation
KMT2A	Lysine Methyltransferase 2A
KMT2A-r	Lysine Methyltransferase 2A Rearrangement
LMR	Low Molecular Risk
LSC score	Leukemic Stem Cell Score
MACE	Amsacrine, Cytarabine and Etoposide
MDS	Myelodysplastic Syndrome
MICE	Mitoxantrone, Cytarabine, and Etoposide
MidAC	Mitoxantrone and Cytarabine
MRC	Medical Research Council
MRD	Minimal Residual Disease
MRP1	Multidrug Resistance-Associated Protein 1
NCRI	National Cancer Research Institute
NPM1	Nucleophosmin 1 gene
NS	Not Significant
ORR	Overall Response Rate
OS	Overall Survival
Pgp	Permeability Glycoprotein
Q	Quartile
RFS	Relapse-Free Survival
RQ-PCR	Real-Time Quantitative Polymerase Chain Reaction
RR	Risk Of Relapse
SHP	Src Homology-2 Domain-Containing Tyrosine Phosphatases
Siglec	Sialic-Acid-Binding Immunoglobulin-Like Lectins Family
SNP	Single Nucleotide Polymorphism
SOCS3	Suppressor Of Cytokine Signaling 3
SWOG	Southwest Oncology Group
TP53	Tumor Protein 53
Vs	Versus
WT1	Wilms’ Tumor 1 Gene

## References

[1] Othus M., Kantarjian H., Petersdorf S., Ravandi F., Godwin J., Cortes J., Pierce S., Erba H., Faderl S., Appelbaum F.R. (2014). Declining rates of treatment-related mortality in patients with newly diagnosed AML given “intense” induction regimens: A report from SWOG and MD anderson. Leukemia.

[2] Cowan A.J., Laszlo G.S., Estey E.H., Walter R.B. (2013). Antibody-based therapy of acute myeloid leukemia with gemtuzumab ozogamicin. Front. Biosci..

[3] Paul S.P., Taylor L.S., Stansbury E.K., McVicar D.W. (2000). Myeloid specific human CD33 is an inhibitory receptor with differential ITIM function in recruiting the phosphatases SHP-1 and SHP-2. Blood.

[4] Godwin C.D., Gale R.P., Walter R.B. (2017). Gemtuzumab ozogamicin in acute myeloid leukemia. Leukemia.

[5] Taylor V.C., Buckley C.D., Douglas M., Cody A.J., Simmons D.L., Freeman S.D. (1999). The myeloid-specific sialic acid-binding receptor, CD33, associates with the protein-tyrosine phosphatases, SHP-1 and SHP-2. J. Biol. Chem..

[6] Orr S.J., Morgan N.M., Elliott J., Burrows J.F., Scott C.J., McVicar D.W., Johnston J.A. (2007). CD33 responses are blocked by SOCS3 through accelerated proteasomal-mediated turnover. Blood.

[7] Andrews R.G., Takahashi M., Segal G.M., Powell J.S., Bernstein I.D., Singer J.W. (1986). The L4F3 antigen is expressed by unipotent and multipotent colony-forming cells but not by their precursors. Blood.

[8] Dick J.E. (2008). Stem cell concepts renew cancer research. Blood.

[9] Walter R.B., Appelbaum F.R., Estey E.H., Bernstein I.D. (2012). Acute myeloid leukemia stem cells and CD33-targeted immunotherapy. Blood.

[10] Welch J.S., Ley T.J., Link D.C., Miller C.A., Larson D.E., Koboldt D.C., Wartman L.D., Lamprecht T.L., Liu F., Xia J. (2012). The origin and evolution of mutations in acute myeloid leukemia. Cell.

[11] Griffin J.D., Linch D., Sabbath K., Larcom P., Schlossman S.F. (1984). A monoclonal antibody reactive with normal and leukemic human myeloid progenitor cells. Leuk. Res..

[12] Dinndorf P.A., Andrews R.G., Benjamin D., Ridgway D., Wolff L., Bernstein I.D. (1986). Expression of normal myeloid-associated antigens by acute leukemia cells. Blood.

[13] Hamann P.R., Hinman L.M., Hollander I., Beyer C.F., Lindh D., Holcomb R., Hallett W., Tsou H.-R., Upeslacis J., Shochat D. (2002). Gemtuzumab ozogamicin, a potent and selective anti-CD33 antibody-calicheamicin conjugate for treatment of acute myeloid leukemia. Bioconjug. Chem..

[14] van Der Velden V.H., te Marvelde J.G., Hoogeveen P.G., Bernstein I.D., Houtsmuller A.B., Berger M.S., van Dongen J.J. (2001). Targeting of the CD33-calicheamicin immunoconjugate Mylotarg (CMA-676) in acute myeloid leukemia: In vivo and in vitro saturation and internalization by leukemic and normal myeloid cells. Blood.

[15] Hamann P.R., Hinman L.M., Beyer C.F., Lindh D., Upeslacis J., Flowers D.A., Bernstein I. (2002). An anti-CD33 antibody-calicheamicin conjugate for treatment of acute myeloid leukemia. Choice of linker. Bioconjug. Chem..

[16] Elmroth K., Nygren J., Mårtensson S., Ismail I.H., Hammarsten O. (2003). Cleavage of cellular DNA by calicheamicin gamma1. DNA Repair.

[17] Linenberger M.L. (2005). CD33-directed therapy with gemtuzumab ozogamicin in acute myeloid leukemia: Progress in understanding cytotoxicity and potential mechanisms of drug resistance. Leukemia.

[18] Amico D., Barbui A.M., Erba E., Rambaldi A., Introna M., Golay J. (2003). Differential response of human acute myeloid leukemia cells to gemtuzumab ozogamicin in vitro: Role of Chk1 and Chk2 phosphorylation and caspase 3. Blood.

[19] Mårtensson S., Nygren J., Osheroff N., Hammarsten O. (2003). Activation of the DNA-dependent protein kinase by drug-induced and radiation-induced DNA strand breaks. Radiat. Res..

[20] Sullivan N., Lyne L. (1990). Sensitivity of fibroblasts derived from ataxia-telangiectasia patients to calicheamicin γ1I. Mutat. Res. Lett..

[21] Prokop A., Wrasidlo W., Lode H., Herold R., Lang F., Henze G., Dörken B., Wieder T., Daniel P.T. (2003). Induction of apoptosis by enediyne antibiotic calicheamicin thetaII proceeds through a caspase-mediated mitochondrial amplification loop in an entirely Bax-dependent manner. Oncogene.

[22] Haag P., Viktorsson K., Lindberg M.L., Kanter L., Lewensohn R., Stenke L. (2009). Deficient activation of Bak and Bax confers resistance to gemtuzumab ozogamicin-induced apoptotic cell death in AML. Exp. Hematol..

[23] Moore J., Seiter K., Kolitz J., Stock W., Giles F., Kalaycio M., Zenk D., Marcucci G. (2006). A Phase II study of Bcl-2 antisense (oblimersen sodium) combined with gemtuzumab ozogamicin in older patients with acute myeloid leukemia in first relapse. Leuk. Res..

[24] Sievers E.L., Appelbaum F.R., Spielberger R.T., Forman S.J., Flowers D., Smith F.O., Shannon-Dorcy K., Berger M.S., Bernstein I.D. (1999). Selective Ablation of Acute Myeloid Leukemia Using Antibody-Targeted Chemotherapy: A Phase I Study of an Anti-CD33 Calicheamicin ImmunoconjugatePresented in part at the 1997 Annual Meeting of the American Society of Clinical Oncology, Denver, CO; the 1997 European Cancer Conference, Hamburg, Germany; and the 1997 Annual Meeting of the American Society of Hematology, San Diego, CA. Blood.

[25] Sievers E.L., Larson R.A., Stadtmauer E.A., Estey E., Löwenberg B., Dombret H., Karanes C., Theobald M., Bennett J.M., Sherman M.L. (2001). Efficacy and safety of gemtuzumab ozogamicin in patients with CD33-positive acute myeloid leukemia in first relapse. J. Clin. Oncol. Off. J. Am. Soc. Clin. Oncol..

[26] Taksin A.-L., Legrand O., Raffoux E., de Revel T., Thomas X., Contentin N., Bouabdallah R., Pautas C., Turlure P., Reman O. (2007). High efficacy and safety profile of fractionated doses of Mylotarg as induction therapy in patients with relapsed acute myeloblastic leukemia: A prospective study of the alfa group. Leukemia.

[27] Farhat H., Reman O., Raffoux E., Berthon C., Pautas C., Kammoun L., Chantepie S., Gardin C., Rousselot P., Chevret S. (2012). Fractionated doses of gemtuzumab ozogamicin with escalated doses of daunorubicin and cytarabine as first acute myeloid leukemia salvage in patients aged 50–70-year old: A phase 1/2 study of the acute leukemia French association. Am. J. Hematol..

[28] Burnett A.K., Hills R.K., Milligan D., Kjeldsen L., Kell J., Russell N.H., Yin J.A.L., Hunter A., Goldstone A.H., Wheatley K. (2011). Identification of patients with acute myeloblastic leukemia who benefit from the addition of gemtuzumab ozogamicin: Results of the MRC AML15 trial. J. Clin. Oncol. Off. J. Am. Soc. Clin. Oncol..

[29] Petersdorf S.H., Kopecky K.J., Slovak M., Willman C., Nevill T., Brandwein J., Larson R.A., Erba H.P., Stiff P.J., Stuart R.K. (2013). A phase 3 study of gemtuzumab ozogamicin during induction and postconsolidation therapy in younger patients with acute myeloid leukemia. Blood.

[30] Burnett A.K., Russell N.H., Hills R.K., Kell J., Freeman S., Kjeldsen L., Hunter A.E., Yin J., Craddock C.F., Dufva I.H. (2012). Addition of gemtuzumab ozogamicin to induction chemotherapy improves survival in older patients with acute myeloid leukemia. J. Clin. Oncol. Off. J. Am. Soc. Clin. Oncol..

[31] Delaunay J., Recher C., Pigneux A., Witz F., Vey N., Blanchet O., Lefebvre P., Luquet I., Guillerme I., Volteau C. (2011). Addition of gemtuzumab ozogamycin to chemotherapy improves event-free survival but not overall survival of AML patients with intermediate cytogenetics not eligible for allogeneic transplantation. results of the GOELAMS AML 2006 IR study. Blood.

[32] Castaigne S., Pautas C., Terré C., Raffoux E., Bordessoule D., Bastie J.-N., Legrand O., Thomas X., Turlure P., Reman O. (2012). Effect of gemtuzumab ozogamicin on survival of adult patients with de-novo acute myeloid leukaemia (ALFA-0701): A randomised, open-label, phase 3 study. Lancet Lond. Engl..

[33] Lambert J., Pautas C., Terré C., Raffoux E., Turlure P., Caillot D., Legrand O., Thomas X., Gardin C., Gogat-Marchant K. (2019). Gemtuzumab ozogamicin for de novo acute myeloid leukemia: Final efficacy and safety updates from the open-label, phase III ALFA-0701 trial. Haematologica.

[34] Burnett A., Cavenagh J., Russell N., Hills R., Kell J., Jones G., Nielsen O.J., Khwaja A., Thomas I., Clark R. (2016). Defining the dose of gemtuzumab ozogamicin in combination with induction chemotherapy in acute myeloid leukemia: A comparison of 3 mg/m2 with 6 mg/m2 in the NCRI AML17 trial. Haematologica.

[35] Amadori S., Suciu S., Stasi R., Salih H.R., Selleslag D., Muus P., De Fabritiis P., Venditti A., Ho A.D., Lübbert M. (2013). Sequential combination of gemtuzumab ozogamicin and standard chemotherapy in older patients with newly diagnosed acute myeloid leukemia: Results of a randomized phase III trial by the EORTC and GIMEMA consortium (AML-17). J. Clin. Oncol. Off. J. Am. Soc. Clin. Oncol..

[36] Amadori S., Suciu S., Selleslag D., Aversa F., Gaidano G., Musso M., Annino L., Venditti A., Voso M.T., Mazzone C. (2016). Gemtuzumab ozogamicin versus best supportive care in older patients with newly diagnosed acute myeloid leukemia unsuitable for intensive chemotherapy: Results of the randomized phase III EORTC-GIMEMA AML-19 trial. J. Clin. Oncol..

[37] Arceci R.J., Sande J., Lange B., Shannon K., Franklin J., Hutchinson R., Vik T.A., Flowers D., Aplenc R., Berger M.S. (2005). Safety and efficacy of gemtuzumab ozogamicin in pediatric patients with advanced CD33+ acute myeloid leukemia. Blood.

[38] Aplenc R., Alonzo T.A., Gerbing R.B., Lange B.J., Hurwitz C.A., Wells R.J., Bernstein I., Buckley P., Krimmel K., Smith F.O. (2008). Safety and efficacy of gemtuzumab ozogamicin in combination with chemotherapy for pediatric acute myeloid leukemia: A report from the children’s oncology group. J. Clin. Oncol. Off. J. Am. Soc. Clin. Oncol..

[39] Cooper T.M., Franklin J., Gerbing R.B., Alonzo T.A., Hurwitz C., Raimondi S.C., Hirsch B., Smith F.O., Mathew P., Arceci R.J. (2012). AAML03P1, a pilot study of the safety of gemtuzumab ozogamicin in combination with chemotherapy for newly diagnosed childhood acute myeloid leukemia: A report from the children’s oncology group. Cancer.

[40] Gamis A.S., Alonzo T.A., Meshinchi S., Sung L., Gerbing R.B., Raimondi S.C., Hirsch B.A., Kahwash S.B., Heerema-McKenney A., Winter L. (2014). Gemtuzumab ozogamicin in children and adolescents with de novo acute myeloid leukemia improves event-free survival by reducing relapse risk: Results from the randomized phase III children’s oncology group trial AAML0531. J. Clin. Oncol. Off. J. Am. Soc. Clin. Oncol..

[41] Bross P.F., Beitz J., Chen G., Chen X.H., Duffy E., Kieffer L., Roy S., Sridhara R., Rahman A., Williams G. (2001). Approval summary: Gemtuzumab ozogamicin in relapsed acute myeloid leukemia. Clin. Cancer Res..

[42] Larson R.A., Sievers E.L., Stadtmauer E.A., Löwenberg B., Estey E.H., Dombret H., Theobald M., Voliotis D., Bennett J.M., Richie M. (2005). Final report of the efficacy and safety of gemtuzumab ozogamicin (Mylotarg) in patients with CD33-positive acute myeloid leukemia in first recurrence. Cancer.

[43] Amadori S., Suciu S., Selleslag D., Stasi R., Alimena G., Baila L., Rizzoli V., Borlenghi E., Gaidano G., Magro D. (2010). Randomized trial of two schedules of low-dose gemtuzumab ozogamicin as induction monotherapy for newly diagnosed acute myeloid leukaemia in older patients not considered candidates for intensive chemotherapy. A phase II study of the EORTC and GIMEMA leukaemia groups (AML-19). Br. J. Haematol..

[44] Kell W.J., Burnett A.K., Chopra R., Yin J.A.L., Clark R.E., Rohatiner A., Culligan D., Hunter A., Prentice A.G., Milligan D.W. (2003). A feasibility study of simultaneous administration of gemtuzumab ozogamicin with intensive chemotherapy in induction and consolidation in younger patients with acute myeloid leukemia. Blood.

[45] Pilorge S., Rigaudeau S., Rabian F., Sarkozy C., Taksin A.L., Farhat H., Merabet F., Ghez S., Raggueneau V., Terré C. (2014). Fractionated gemtuzumab ozogamicin and standard dose cytarabine produced prolonged second remissions in patients over the age of 55 years with acute myeloid leukemia in late first relapse. Am. J. Hematol..

[46] Hills R.K., Castaigne S., Appelbaum F.R., Delaunay J., Petersdorf S., Othus M., Estey E.H., Dombret H., Chevret S., Ifrah N. (2014). Addition of gemtuzumab ozogamicin to induction chemotherapy in adult patients with acute myeloid leukaemia: A meta-analysis of individual patient data from randomised controlled trials. Lancet Oncol..

[47] Pollard J.A., Alonzo T.A., Loken M., Gerbing R.B., Ho P.A., Bernstein I.D., Raimondi S.C., Hirsch B., Franklin J., Walter R.B. (2012). Correlation of CD33 expression level with disease characteristics and response to gemtuzumab ozogamicin containing chemotherapy in childhood AML. Blood.

[48] Pollard J.A., Loken M., Gerbing R.B., Raimondi S.C., Hirsch B.A., Aplenc R., Bernstein I.D., Gamis A.S., Alonzo T.A., Meshinchi S. (2016). CD33 expression and its association with gemtuzumab ozogamicin response: Results from the randomized phase III children’s oncology group trial AAML0531. J. Clin. Oncol. Off. J. Am. Soc. Clin. Oncol..

[49] Dinndorf P.A., Buckley J.D., Nesbit M.E., Lampkin B.C., Piomelli S., Feig S.A., Kersey J.H., Hammond G.D., Bernstein I.D. (1992). Expression of myeloid differentiation antigens in acute nonlymphocytic leukemia: Increased concentration of CD33 antigen predicts poor outcome—A report from the childrens cancer study group. Med. Pediatr. Oncol..

[50] Walter R.B., Raden B.W., Kamikura D.M., Cooper J.A., Bernstein I.D. (2005). Influence of CD33 expression levels and ITIM-dependent internalization on gemtuzumab ozogamicin-induced cytotoxicity. Blood.

[51] Walter R.B., Gooley T.A., van der Velden V.H.J., Loken M.R., van Dongen J.J.M., Flowers D.A., Bernstein I.D., Appelbaum F.R. (2007). CD33 expression and P-glycoprotein-mediated drug efflux inversely correlate and predict clinical outcome in patients with acute myeloid leukemia treated with gemtuzumab ozogamicin monotherapy. Blood.

[52] Khan N., Hills R.K., Virgo P., Couzens S., Clark N., Gilkes A., Richardson P., Knapper S., Grimwade D., Russell N.H. (2017). Expression of CD33 is a predictive factor for effect of gemtuzumab ozogamicin at different doses in adult acute myeloid leukaemia. Leukemia.

[53] De Propris M.S., Raponi S., Diverio D., Milani M.L., Meloni G., Falini B., Foà R., Guarini A. (2011). High CD33 expression levels in acute myeloid leukemia cells carrying the nucleophosmin (NPM1) mutation. Haematologica.

[54] Ehninger A., Kramer M., Röllig C., Thiede C., Bornhäuser M., von Bonin M., Wermke M., Feldmann A., Bachmann M., Ehninger G. (2014). Distribution and levels of cell surface expression of CD33 and CD123 in acute myeloid leukemia. Blood Cancer J..

[55] Olombel G., Guerin E., Guy J., Perrot J.-Y., Dumezy F., de Labarthe A., Bastie J.-N., Legrand O., Raffoux E., Plesa A. (2016). The level of blast CD33 expression positively impacts the effect of gemtuzumab ozogamicin in patients with acute myeloid leukemia. Blood.

[56] Renneville A., Abdelali R.B., Chevret S., Nibourel O., Cheok M., Pautas C., Duléry R., Boyer T., Cayuela J.-M., Hayette S. (2014). Clinical impact of gene mutations and lesions detected by SNP-array karyotyping in acute myeloid leukemia patients in the context of gemtuzumab ozogamicin treatment: Results of the ALFA-0701 trial. Oncotarget.

[57] Wiemels J.L., Xiao Z., Buffler P.A., Maia A.T., Ma X., Dicks B.M., Smith M.T., Zhang L., Feusner J., Wiencke J. (2002). In utero origin of t(8;21) AML1-ETO translocations in childhood acute myeloid leukemia. Blood.

[58] Jourdan E., Boissel N., Chevret S., Delabesse E., Renneville A., Cornillet P., Blanchet O., Cayuela J.-M., Recher C., Raffoux E. (2013). Prospective evaluation of gene mutations and minimal residual disease in patients with core binding factor acute myeloid leukemia. Blood.

[59] Appelbaum F.R., Bernstein I.D. (2017). Gemtuzumab ozogamicin for acute myeloid leukemia. Blood.

[60] Borthakur G., Cortes J.E., Estey E.E., Jabbour E., Faderl S., O′Brien S., Garcia-Manero G., Kadia T.M., Wang X., Patel K. (2014). Gemtuzumab ozogamicin with fludarabine, cytarabine, and granulocyte colony stimulating factor (FLAG-GO) as front-line regimen in patients with core binding factor acute myelogenous leukemia. Am. J. Hematol..

[61] Borthakur G.M., Cortes J.E., Ravandi F., Garcia-Manero G., Kadia T.M., Jabbour E., Patel K., Issa G.C., Daver N.G., Ohanian M.N. (2019). Fludarabine, cytarabine, G-CSF and gemtuzumab ozogamicin (FLAG-GO) regimen results in better molecular response and relapse-free survival in core binding factor acute myeloid leukemia than FLAG and idarubicin (FLAG-Ida). Blood.

[62] Lo-Coco F., Avvisati G., Vignetti M., Thiede C., Orlando S.M., Iacobelli S., Ferrara F., Fazi P., Cicconi L., Di Bona E. (2013). Retinoic Acid and Arsenic Trioxide for Acute Promyelocytic Leukemia. N. Engl. J. Med..

[63] Sanz M.A., Fenaux P., Tallman M.S., Estey E.H., Löwenberg B., Naoe T., Lengfelder E., Döhner H., Burnett A.K., Chen S.-J. (2019). Management of acute promyelocytic leukemia: Updated recommendations from an expert panel of the European leukemianet. Blood.

[64] Michieli M., Damiani D., Ermacora A., Geromin A., Michelutti A., Masolini P., Baccarani M. (2000). P-glycoprotein (PGP), lung resistance-related protein (LRP) and multidrug resistance-associated protein (MRP) expression in acute promyelocytic leukaemia. Br. J. Haematol..

[65] Takeshita A., Shinjo K., Naito K., Matsui H., Sahara N., Shigeno K., Horii T., Shirai N., Maekawa M., Ohnishi K. (2005). Efficacy of gemtuzumab ozogamicin on ATRA- and arsenic-resistant acute promyelocytic leukemia (APL) cells. Leukemia.

[66] Breccia M., Lo-Coco F. (2011). Gemtuzumab ozogamicin for the treatment of acute promyelocytic leukemia: Mechanisms of action and resistance, safety and efficacy. Expert Opin. Biol. Ther..

[67] Estey E.H., Giles F.J., Beran M., O’Brien S., Pierce S.A., Faderl S.H., Cortes J.E., Kantarjian H.M. (2002). Experience with gemtuzumab ozogamycin (“mylotarg”) and all-trans retinoic acid in untreated acute promyelocytic leukemia. Blood.

[68] Lo-Coco F., Cimino G., Breccia M., Noguera N.I., Diverio D., Finolezzi E., Pogliani E.M., Di Bona E., Micalizzi C., Kropp M. (2004). Gemtuzumab ozogamicin (Mylotarg) as a single agent for molecularly relapsed acute promyelocytic leukemia. Blood.

[69] Breccia M., Cimino G., Diverio D., Gentilini F., Mandelli F., Lo Coco F. (2007). Sustained molecular remission after low dose gemtuzumab-ozogamicin in elderly patients with advanced acute promyelocytic leukemia. Haematologica.

[70] Burnett A.K., Russell N.H., Hills R.K., Bowen D., Kell J., Knapper S., Morgan Y.G., Lok J., Grech A., Jones G. (2015). Arsenic trioxide and all-trans retinoic acid treatment for acute promyelocytic leukaemia in all risk groups (AML17): Results of a randomised, controlled, phase 3 trial. Lancet Oncol..

[71] Takeshita A., Shinjo K., Naito K., Matsui H., Sahara N., Shigeno K., Suzumura T., Horii T., Shirai N., Maekawa M. (2005). Two patients with all-trans retinoic acid-resistant acute promyelocytic leukemia treated successfully with gemtuzumab ozogamicin as a single agent. Int. J. Hematol..

[72] Ravandi F., Estey E., Jones D., Faderl S., O′Brien S., Fiorentino J., Pierce S., Blamble D., Estrov Z., Wierda W. (2009). Effective treatment of acute promyelocytic leukemia with all-trans-retinoic acid, arsenic trioxide, and gemtuzumab ozogamicin. J. Clin. Oncol. Off. J. Am. Soc. Clin. Oncol..

[73] Abaza Y., Kantarjian H., Garcia-Manero G., Estey E., Borthakur G., Jabbour E., Faderl S., O′Brien S., Wierda W., Pierce S. (2017). Long-term outcome of acute promyelocytic leukemia treated with all-trans-retinoic acid, arsenic trioxide, and gemtuzumab. Blood.

[74] Lancet J.E., Moseley A.B., Coutre S.E., DeAngelo D.J., Othus M., Tallman M.S., Litzow M.R., Komrokji R.S., Erba H.P., Appelbaum F.R. (2020). A phase 2 study of ATRA, arsenic trioxide, and gemtuzumab ozogamicin in patients with high-risk APL (SWOG 0535). Blood Adv..

[75] Muñoz L., Nomdedéu J.F., Villamor N., Guardia R., Colomer D., Ribera J.M., Torres J.P., Berlanga J.J., Fernández C., Llorente A. (2003). Acute myeloid leukemia with MLL rearrangements: Clinicobiological features, prognostic impact and value of flow cytometry in the detection of residual leukemic cells. Leukemia.

[76] Tamai H., Shioi Y., Yamaguchi H., Okabe M., Wakita S., Mizuki T., Nakayama K., Inokuchi K., Tajika K., Dan K. (2008). Treatment of relapsed acute myeloid leukemia with MLL/AF6 fusion after allogeneic hematopoietic stem cell transplantation with gemtuzumab ozogamicin with a long interval followed by donor lymphocyte infusion. Leukemia.

[77] Asano H., Yamamoto G., Hosoi M., Takahashi T., Hangaishi A., Kurokawa M. (2010). Complete molecular remission in refractory acute myeloid leukemia with MLL/AF9 treated with gemtuzumab ozogamicin. Leuk. Res..

[78] Pollard J., Alonzo T.A., Gerbing R.B., Raimondi S.C., Hirsch B.A., Sung L., Aplenc R., Guest E.M., Bernstein I.D., Loken M.R. (2015). Treatment of 11q23/MLL + AML with gemtuzumab ozogamicin: Results from the randomized phase III children’s oncology group trial AAML0531. Blood.

[79] Döhner H., Weisdorf D.J., Bloomfield C.D. (2015). Acute myeloid leukemia. N. Engl. J. Med..

[80] Schlenk R.F., Paschka P., Krzykalla J., Weber D., Kapp-Schwoerer S., Gaidzik V.I., Leis C., Fiedler W., Kindler T., Schroeder T. (2020). Gemtuzumab ozogamicin in NPM1-mutated acute myeloid leukemia: Early results from the prospective randomized AMLSG 09-09 phase III study. J. Clin. Oncol. Off. J. Am. Soc. Clin. Oncol..

[81] Tarlock K., Alonzo T.A., Gerbing R.B., Raimondi S.C., Hirsch B.A., Sung L., Pollard J.A., Aplenc R., Loken M.R., Gamis A.S. (2016). Gemtuzumab ozogamicin reduces relapse risk in FLT3/ITD acute myeloid leukemia: A report from the children’s oncology group. Clin. Cancer Res. Off. J. Am. Assoc. Cancer Res..

[82] Papaemmanuil E., Gerstung M., Bullinger L., Gaidzik V.I., Paschka P., Roberts N.D., Potter N.E., Heuser M., Thol F., Bolli N. (2016). Genomic classification and prognosis in acute myeloid leukemia. N. Engl. J. Med..

[83] Döhner H., Estey E., Grimwade D., Amadori S., Appelbaum F.R., Büchner T., Dombret H., Ebert B.L., Fenaux P., Larson R.A. (2017). Diagnosis and management of AML in adults: 2017 ELN recommendations from an international expert panel. Blood.

[84] Fournier E., Duployez N., Ducourneau B., Raffoux E., Turlure P., Caillot D., Thomas X., Marceau-Renaut A., Chantepie S., Malfuson J.-V. (2020). Mutational profile and benefit of gemtuzumab ozogamicin in acute myeloid leukemia. Blood.

[85] Freeman S.D., Virgo P., Couzens S., Grimwade D., Russell N., Hills R.K., Burnett A.K. (2013). Prognostic relevance of treatment response measured by flow cytometric residual disease detection in older patients with acute myeloid leukemia. J. Clin. Oncol. Off. J. Am. Soc. Clin. Oncol..

[86] Freeman S.D., Hills R.K., Virgo P., Khan N., Couzens S., Dillon R., Gilkes A., Upton L., Nielsen O.J., Cavenagh J.D. (2018). Measurable residual disease at induction redefines partial response in acute myeloid leukemia and stratifies outcomes in patients at standard risk without NPM1 mutations. J. Clin. Oncol..

[87] Chen X., Xie H., Wood B.L., Walter R.B., Pagel J.M., Becker P.S., Sandhu V.K., Abkowitz J.L., Appelbaum F.R., Estey E.H. (2015). Relation of clinical response and minimal residual disease and their prognostic impact on outcome in acute myeloid leukemia. J. Clin. Oncol..

[88] Walter R.B., Gyurkocza B., Storer B.E., Godwin C.D., Pagel J.M., Buckley S.A., Sorror M.L., Wood B.L., Storb R., Appelbaum F.R. (2015). Comparison of minimal residual disease as outcome predictor for AML patients in first complete remission undergoing myeloablative or nonmyeloablative allogeneic hematopoietic cell transplantation. Leukemia.

[89] Balsat M., Renneville A., Thomas X., de Botton S., Caillot D., Marceau A., Lemasle E., Marolleau J.-P., Nibourel O., Berthon C. (2017). Postinduction minimal residual disease predicts outcome and benefit from allogeneic stem cell transplantation in acute myeloid leukemia with NPM1 mutation: A study by the acute leukemia french association group. J. Clin. Oncol..

[90] Jongen-Lavrencic M., Grob T., Hanekamp D., Kavelaars F.G., al Hinai A., Zeilemaker A., Erpelinck-Verschueren C.A.J., Gradowska P.L., Meijer R., Cloos J. (2018). Molecular minimal residual disease in acute myeloid leukemia. N. Engl. J. Med..

[91] Hourigan C.S., Dillon L.W., Gui G., Logan B.R., Fei M., Ghannam J., Li Y., Licon A., Alyea E.P., Bashey A. (2020). Impact of conditioning intensity of allogeneic transplantation for acute myeloid leukemia with genomic evidence of residual disease. J. Clin. Oncol. Off. J. Am. Soc. Clin. Oncol..

[92] Schuurhuis G.J., Heuser M., Freeman S., Béné M.-C., Buccisano F., Cloos J., Grimwade D., Haferlach T., Hills R.K., Hourigan C.S. (2018). Minimal/measurable residual disease in AML: A consensus document from the European leukemianet MRD working party. Blood.

[93] Rubnitz J.E., Inaba H., Dahl G., Ribeiro R.C., Bowman W.P., Taub J., Pounds S., Razzouk B.I., Lacayo N.J., Cao X. (2010). Minimal residual disease-directed therapy for childhood acute myeloid leukaemia: Results of the AML02 multicentre trial. Lancet Oncol..

[94] O′Hear C., Inaba H., Pounds S., Shi L., Dahl G., Bowman W.P., Taub J.W., Pui C.-H., Ribeiro R.C., Coustan-Smith E. (2013). Gemtuzumab ozogamicin can reduce minimal residual disease in patients with childhood acute myeloid leukemia. Cancer.

[95] Lapillonne H., Renneville A., Auvrignon A., Flamant C., Blaise A., Perot C., Lai J.-L., Ballerini P., Mazingue F., Fasola S. (2006). High WT1 expression after induction therapy predicts high risk of relapse and death in pediatric acute myeloid leukemia. J. Clin. Oncol. Off. J. Am. Soc. Clin. Oncol..

[96] Cilloni D., Renneville A., Hermitte F., Hills R.K., Daly S., Jovanovic J.V., Gottardi E., Fava M., Schnittger S., Weiss T. (2009). Real-time quantitative polymerase chain reaction detection of minimal residual disease by standardized WT1 assay to enhance risk stratification in acute myeloid leukemia: A European Leukemianet study. J. Clin. Oncol. Off. J. Am. Soc. Clin. Oncol..

[97] Lambert J., Lambert J., Nibourel O., Pautas C., Hayette S., Cayuela J.-M., Terré C., Rousselot P., Dombret H., Chevret S. (2014). MRD assessed by WT1 and NPM1 transcript levels identifies distinct outcomes in AML patients and is influenced by gemtuzumab ozogamicin. Oncotarget.

[98] Ivey A., Hills R.K., Simpson M.A., Jovanovic J.V., Gilkes A., Grech A., Patel Y., Bhudia N., Farah H., Mason J. (2016). Assessment of minimal residual disease in standard-risk AML. N. Engl. J. Med..

[99] Kayser S., Benner A., Thiede C., Martens U., Huber J., Stadtherr P., Janssen J.W.G., Röllig C., Uppenkamp M.J., Bochtler T. (2016). Pretransplant NPM1 MRD levels predict outcome after allogeneic hematopoietic stem cell transplantation in patients with acute myeloid leukemia. Blood Cancer J..

[100] Candoni A., De Marchi F., Zannier M.E., Lazzarotto D., Filì C., Dubbini M.V., Rabassi N., Toffoletti E., Lau B.W., Fanin R. (2017). High prognostic value of pre-allogeneic stem cell transplantation minimal residual disease detection by WT1 gene expression in AML transplanted in cytologic complete remission. Leuk. Res..

[101] Dillon R., Hills R., Freeman S., Potter N., Jovanovic J., Ivey A., Kanda A.S., Runglall M., Foot N., Valganon M. (2020). Molecular MRD status and outcome after transplantation in NPM1-mutated AML. Blood.

[102] Ball B., Stein E.M. (2019). Which are the most promising targets for minimal residual disease-directed therapy in acute myeloid leukemia prior to allogeneic stem cell transplant?. Haematologica.

[103] Bonnet D., Dick J.E. (1997). Human acute myeloid leukemia is organized as a hierarchy that originates from a primitive hematopoietic cell. Nat. Med..

[104] Thomas D., Majeti R. (2017). Biology and relevance of human acute myeloid leukemia stem cells. Blood.

[105] Ng S.W.K., Mitchell A., Kennedy J.A., Chen W.C., McLeod J., Ibrahimova N., Arruda A., Popescu A., Gupta V., Schimmer A.D. (2016). A 17-gene stemness score for rapid determination of risk in acute leukaemia. Nature.

[106] Ng S.W.K., Erwin S.E., Mitchell A., Minden M.D., Bullinger L., Döhner H., Dombret H., Preudhomme C., Cheok M., Dick J.E. (2018). A novel predictor of response to gemtuzumab ozogamicin therapy in AML provides strategies for sensitization of leukemia stem cells in individual patients. Blood.

[107] Lamba J.K., Pounds S., Cao X., Downing J.R., Campana D., Ribeiro R.C., Pui C.-H., Rubnitz J.E. (2009). Coding polymorphisms in CD33 and response to gemtuzumab ozogamicin in pediatric patients with AML: A pilot study. Leukemia.

[108] Raj T., Ryan K.J., Replogle J.M., Chibnik L.B., Rosenkrantz L., Tang A., Rothamel K., Stranger B.E., Bennett D.A., Evans D.A. (2014). CD33: Increased inclusion of exon 2 implicates the Ig V-set domain in alzheimer’s disease susceptibility. Hum. Mol. Genet..

[109] Malik M., Chiles J., Xi H.S., Medway C., Simpson J., Potluri S., Howard D., Liang Y., Paumi C.M., Mukherjee S. (2015). Genetics of CD33 in alzheimer’s disease and acute myeloid leukemia. Hum. Mol. Genet..

[110] Mortland L., Alonzo T.A., Walter R.B., Gerbing R.B., Mitra A.K., Pollard J.A., Loken M.R., Hirsch B., Raimondi S., Franklin J. (2013). Clinical significance of CD33 nonsynonymous single-nucleotide polymorphisms in pediatric patients with acute myeloid leukemia treated with gemtuzumab-ozogamicin-containing chemotherapy. Clin. Cancer Res. Off. J. Am. Assoc. Cancer Res..

[111] Lamba J.K., Chauhan L., Shin M., Loken M.R., Pollard J.A., Wang Y.-C., Ries R.E., Aplenc R., Hirsch B.A., Raimondi S.C. (2017). CD33 splicing polymorphism determines gemtuzumab ozogamicin response in de novo acute myeloid leukemia: Report from randomized phase III children’s oncology group trial AAML0531. J. Clin. Oncol..

[112] Gale R.E., Popa T., Wright M., Khan N., Freeman S.D., Burnett A.K., Russell N.H., Hills R.K., Linch D.C. (2018). No evidence that CD33 splicing SNP impacts the response to GO in younger adults with AML treated on UK MRC/NCRI trials. Blood.

[113] Stanchina M., Pastore A., Devlin S., Famulare C., Stein E., Taylor J. (2019). CD33 splice site genotype was not associated with outcomes of patients receiving the anti-CD33 drug conjugate SGN-CD33A. J. Hematol. Oncol..

[114] Leith C.P., Kopecky K.J., Chen I.M., Eijdems L., Slovak M.L., McConnell T.S., Head D.R., Weick J., Grever M.R., Appelbaum F.R. (1999). Frequency and clinical significance of the expression of the multidrug resistance proteins MDR1/P-glycoprotein, MRP1, and LRP in acute myeloid leukemia: A southwest oncology group study. Blood.

[115] Laszlo G.S., Beddoe M.E., Godwin C.D., Bates O.M., Gudgeon C.J., Harrington K.H., Walter R.B. (2019). Relationship between CD33 expression, splicing polymorphism, and in vitro cytotoxicity of gemtuzumab ozogamicin and the CD33/CD3 BiTE ^®^ AMG 330. Haematologica.

[116] Chauhan L., Shin M., Wang Y.-C., Loken M., Pollard J., Aplenc R., Hirsch B.A., Raimondi S., Ries R.E., Bernstein I.D. (2019). CD33_PGx6_score predicts gemtuzumab ozogamicin response in childhood acute myeloid leukemia: A report from the children’s oncology group. JCO Precis. Oncol..

[117] Linenberger M.L., Hong T., Flowers D., Sievers E.L., Gooley T.A., Bennett J.M., Berger M.S., Leopold L.H., Appelbaum F.R., Bernstein I.D. (2001). Multidrug-resistance phenotype and clinical responses to gemtuzumab ozogamicin. Blood.

[118] Walter R.B., Raden B.W., Hong T.C., Flowers D.A., Bernstein I.D., Linenberger M.L. (2003). Multidrug resistance protein attenuates gemtuzumab ozogamicin-induced cytotoxicity in acute myeloid leukemia cells. Blood.

[119] Walter R.B., Raden B.W., Thompson J., Flowers D.A., Kiem H.-P., Bernstein I.D., Linenberger M.L. (2004). Breast cancer resistance protein (BCRP/ABCG2) does not confer resistance to gemtuzumab ozogamicin and calicheamicin-gamma1 in acute myeloid leukemia cells. Leukemia.

[120] Boyer T., Gonzales F., Barthélémy A., Marceau-Renaut A., Peyrouze P., Guihard S., Lepelley P., Plesa A., Nibourel O., Delattre C. (2019). Clinical significance of ABCB1 in acute myeloid leukemia: A comprehensive study. Cancers.

[121] Van Den Heuvel-Eibrink M.M., Van Der Holt B., Te Boekhorst P.A., Pieters R., Schoester M., Löwenberg B., Sonneveld P. (1997). MDR 1 expression is an independent prognostic factor for response and survival in de novo acute myeloid leukaemia. Br. J. Haematol..

[122] Rafiee R., Chauhan L., Alonzo T.A., Wang Y.-C., Elmasry A., Loken M.R., Pollard J., Aplenc R., Raimondi S., Hirsch B.A. (2019). ABCB1 SNP predicts outcome in patients with acute myeloid leukemia treated with gemtuzumab ozogamicin: A report from children’s oncology group AAML0531 trial. Blood Cancer J..

[123] Ball E.D. (2007). Pairing SOCS with CD33. Blood.

[124] Middeldorf I., Galm O., Osieka R., Jost E., Herman J.G., Wilop S. (2010). Sequence of administration and methylation of SOCS3 may govern response to gemtuzumab ozogamicin in combination with conventional chemotherapy in patients with refractory or relapsed acute myelogenous leukemia (AML). Am. J. Hematol..

[125] Paubelle E., Marceau A., Zylbersztejn F., Dussiot M., Moura I.C., Cornillet-Lefebvre P., Delaunay J., Burnett A.K., Castaigne S., Guardiola P. (2015). HFE gene mutation status predicts response to gemtuzumab ozogamicin in AML. Blood.

